# An Empirical Analysis of Social Interaction in Online Teaching in Open Universities Based on Flipped Classroom

**DOI:** 10.1155/2022/3089239

**Published:** 2022-10-11

**Authors:** Xinjie Wang, Ding Yuan

**Affiliations:** ^1^College of Humanities and Education, Beijing Open University, Beijing, China; ^2^Beijing Mycourse Online Educational Technology Co. Ltd, Beijing, China

## Abstract

As a teaching model, flipped classroom can stimulate students' learning interests and promote students' positive social interactions. As an adult university relying on information technology to carry out online teaching, open universities need to pay attention to the online learning quality of adult students. This study attempts to apply the concept of flipped classroom to online teaching in open universities. While exploring the feasibility of the online flipped classroom teaching model, it adopts an equal group experiment method, content analysis method, and social network analysis method to compare the social interaction level difference between online teaching based on flipped classroom and conventional online teaching. The results show that online flipped classroom has a positive and significant impact on student's cognitive processing level and knowledge construction level and can promote groups to form complex knowledge network forms. This shows that network teaching based on flipped classroom can exercise students' high-order thinking ability, enhance students' cooperative consciousness and behavior, and stimulate students' subjectivity in learning. The purpose of this study is to provide references for promoting interactive behavior and quality in online teaching.

## 1. Study Background

In 2021, the outline of the National 14th Five-Year Plan once again proposed to “give full play to the advantages of online education and build a society in which people enjoy learning.” As a new type of university that leverages information technology to carry out distance education for working adults, open universities are expected to innovate the approaches to facilitation of learning and improve the effectiveness of online teaching (JZC [2016] no.2). In an online learning environment, social interaction is a manifestation of mutual influence and mutual role playing between learners and resources, teachers, and peers. Quality, in-depth interactions are required to enhance the effectiveness of teaching and achieve in-depth learning [1]. However, currently, in online teaching at open universities, desired classroom behaviors such as active collaboration, focused exploration, and in-depth communication among students are seldom seen in the interaction sessions. How to break such a deadlock, promote interactive participation among students, and improve the quality of interaction? This is the vision and need for open universities to improve the quality of online education and teaching.

The flipped classroom originated in the United States. It is designed to establish a classroom centered on students' learning so that classroom-based learning can be upgraded from superficial to in-depth learning and develop the higher-order cognition capacity of students [2]. Studies have shown that flipped classroom can mobilize students to actively participate in classroom activities, facilitate students to learn actively in a collaborative way, and improve their academic performance [3]. Therefore, applying flipped classroom to online teaching at open universities can be expected to enhance the depth and breadth of student interactions, thus improving learning outcomes. In order to validate this hypothesis, this study attempted to design a flipped classroom-based online teaching model and applied it to the course “Kindergarten Management” at Beijing Open University for an empirical study.

## 2. Relevant Studies

### 2.1. Definition of the Concept

#### 2.1.1. Flipped Classroom

There are different interpretations of flipped classroom in Chinese and international academic circles, which highlight different characteristics of flipped classroom. The first interpretation sees flipped classroom as a student-centered teaching approach that emphasizes teachers' use of heuristic, exploratory, and discussion-based teaching methods in the classroom to motivate students to acquire knowledge through collaborative learning and active learning [4]. The second considers flipped classroom as a new learning model realized via information technology that extends conventional classroom activities outside the classroom [5]. The third focuses on the value of flipped classroom in terms of knowledge internalization, which increases the instances of knowledge internalization during active interactions between teachers and students and among students in the classroom based on time series reconstruction, in order to achieve in-depth learning [6]. The above studies, respectively, reveal the vision, methods, environment, and outcomes of teaching through flipped classroom. Generally speaking, the vision for teaching through flipped classroom is student-centered, which can leverage information technology to expand the learning environment and promote students' acquisition and application of in-depth knowledge through appropriate and orderly self-directed and collaborative learning. This study focuses on the design of teaching based on the concept and value of flipped classroom, i.e., the classroom is centered on students' questions, with diverse learning activities such as heuristic, exploratory learning activities, and teachers stimulate the intrinsic motivation for learning by guiding students to communicate and collaborate, share knowledge, and others, ultimately resulting in the teaching process composed of three stages: self-directed basic learning, exploratory in-depth learning, and reflective enhanced learning.

#### 2.1.2. Social Interaction

Social interaction is a key element of distance education, which enables learners to achieve individualized interaction with learning resources and communication with teachers and other learners in the online learning environment [7], which is conducive to the joint development and sharing of learning knowledge, as well as cognitive enhancement [8]. Bates [9] first proposed that social interaction is the interaction of learners with teachers and other learners about learning [9]. Based on Bates' classification theory, Chen Li, a Chinese scholar, further pointed out that social interaction in the field of distance learning means teacher-student information exchange and student-student information exchange [10]. Only those interactions through which learners actively share, collaborate, and enhance their cognition capacity can facilitate effective learning.

Social interaction can be categorized into synchronous interaction and asynchronous interaction according to its timeliness. Asynchronous interaction is the popular form of social interaction for adult learners, which provides learners with a space for discussion that is relatively free in terms of time and space and records the content of discussion thoroughly through the online platform, thus triggering learners' in-depth thinking [11]. In summary, this study focuses on social interaction in an asynchronous interaction environment, which refers to the information exchange and discussion between students, teachers, and peers through asynchronous interaction in a classroom-based learning environment in order to construct knowledge structures relevant to course learning. Specifically, it includes interactive behaviors such as teachers and students expressing their views, asking questions, and giving feedback in a discussion forum on an online learning platform.

### 2.2. Analysis of the Effect of Flipped Classroom Teaching

The core idea of flipped classroom, as a major revolution affecting classroom teaching, is to turn the conventional classroom into an interactive setting. Compared with the conventional teaching model, the model of flipped classroom teaching can have a positive impact on learners' learning performance, cognitive skills, and emotions/attitudes towards learning, which is conducive to improving learning outcomes; the stronger the learners' autonomy, initiative, willpower, and control, the better the learning outcomes [12]. Meanwhile, there are also studies concluding that small-scale teaching and humanities courses can be better taught using flipped classroom [13, 14]. Subsequently, more studies attempted to combine flipped classroom with other teaching models in order to take advantage of their respective strengths to jointly enhance teaching effectiveness. For example, the combination of microlectures and MOOCs with flipped classroom solves the problem of work-learning contradiction among adult learners, enabling them to learn anytime and anywhere, and effectively leverages face-to-face teaching sessions to organize student discussion, which increases teacher-student interactions and improves the efficiency of learning [15, 16]. Besides, the measurement and evaluation of the effectiveness of flipped classroom teaching mostly focus on three aspects, namely, students' learning attitude, learning process, and learning outcome. Learning attitude mainly examines whether students voluntarily and actively participate in learning activities, learning process mainly examines students' development of capability in communication and interaction, collaboration, thinking, and other cognitive skills in the classroom, and learning outcome refers to students' academic performance [1, 12, 17].

There have been studies testifying the applicability and effectiveness of flipped classroom and describing the advantages and effectiveness of flipped classroom. However, the majority of previous studies simply examined face-to-face teaching in primary and secondary schools and regular higher education institutions and seldom focused on online teaching in open universities. Among the few studies that examined teaching in flipped classroom for adult students in open education settings, most simply focused on the design and evaluation of the teaching models, and there are limited empirical studies examining the teaching outcomes.

### 2.3. Associations between Classroom-Based Learning and Social Interaction

According to Vygotsky (1978), learners need to interact with people in their environment in order to awaken a variety of internal developmental processes [18]. Thus, social interaction is an important precondition for learning to take place. The learning theory of social constructionism views learning as an active process that occurs in concrete environments and interpersonal relationships, where knowledge is constructed through language and other social tools and where the meaning of knowledge is verified through discussion and application [19]. Therefore, in classroom-based learning, teacher-student interaction is mainly about students receiving guidance and assistance from the teacher, while student-student interaction is mainly about communication and collaboration in order to complete a certain task [20]. In this process, the more students proactively communicate and collaborate, initiate profound cognitive processing and involvement, and engage in meaningful construction, the more effective the learning will be.

Connectivism learning emphasizes the establishment of personal neural networks, conceptual networks, and social networks, and essentially only complicated connections that can yield knowledge innovations are true connectivism learning [13]. In connectionist classroom-based learning, the teacher, as a node in the overall learning network, plays the role of facilitating, enhancing, and maintaining the classroom environment and knowledge network. Student-student interaction is the process of social network formation in the course of classroom-based learning, followed by the process of collective knowledge innovation. Generally speaking, more complicated network construction and stronger connectivity mean a higher level of students' participation, more knowledge innovation based on such network, and better outcomes of collective learning.

### 2.4. Studies on the Methods for Evaluation of Social Interaction Quality

The evaluation of the quality of social interaction is mostly based on a combination of the content analysis method and the social network analysis method. The content analysis method involves qualitative analysis of the quality of social interaction content [21], which usually leverages a coding scale with multidimensional and multilevel gauges to analyze the quality of the content of interaction to reveal the pattern of interaction [22], including Henri's five-dimensional framework for cognitive learning, Stacy's collaborative learning framework, Gunawardena's interaction analysis model based on the five-phase social construction of knowledge, and others[23]. Bloom's taxonomy of learning objectives is also commonly used to examine the depth of instructional interactive textual content and the learners' cognition process patterns and levels. Social network analysis is a method to analyze the representation and characteristics of social networks by examining the interactive relationships among different actors [24]. UCINET, a social network analysis software with powerful matrix analysis functions and support for generating various visual maps, can effectively reveal the characteristics of the overall interaction network of learners and the roles played by individuals in the network [25].

## 3. Study Design and Methodology

### 3.1. Study Sample

The study sample was composed of 77 working adult students taking the preschool education course “Kindergarten Management” at Beijing Open University, as well as one instructor. All the students were newly enrolled by the university in the fall of 2018, and most were working at kindergartens with some experience in preschool education. The course “Kindergarten Management” was the first specialty course that the students have been exposed to since they were enrolled; relevant professional-level tests were conducted before the class, and the results showed that there was little difference in the student's prior knowledge and experience in the subject and familiarity with the operation of the learning platform. The instructor has been trained in the expertise related to the course and the learning platform and has been tutoring the course for five years.

### 3.2. Study Design

#### 3.2.1. Design of Flipped Classroom Teaching

**Figure 1:**
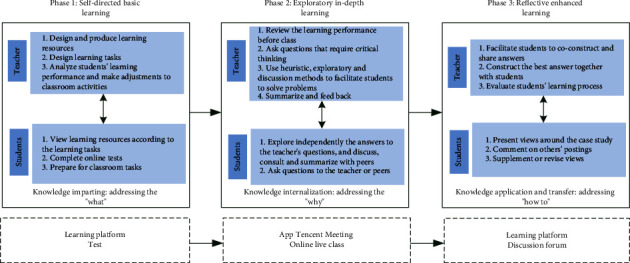
Procedures of online teaching based on flipped classroom.

The course “Kindergarten Management” at Beijing Open University has been implemented with flipped classroom teaching design. The course was developed on the Moodle platform in the fall of 2013 and has been implemented several times since then, with a robust practice basis. The course is taught online for six weeks, with each week being an independent learning module. The learning resources include videos and texts, the learning activities are online discussions, and the learning appraisal approaches include tests, forums, and assignments. Since this study focuses on the quality of students' social interactions, the weeks when forum activities were organized, i.e., the third and sixth weeks, were selected for the implementation of flipped classroom. The design is based on the concept of “student-centered learning” with learning tasks throughout the teaching process and focuses on the internalization in the classroom through three phases of teaching, namely, “self-directed basic learning,” “exploratory in-depth learning,” and “reflective enhanced learning.” In such a design, superficial knowledge precedes in-depth knowledge, and the in-depth knowledge is taught through heuristic dialogue activities and exploratory extension activities. The specific operating procedures are shown in Figure 1.

Phase 1: self-directed basic learning, focusing on students' acquisition of superficial knowledge. The teacher presents the learning tasks and timeline for the module before the start of the course module. On the course learning platform, the teacher designs and develops relevant learning resources, including video and text learning materials, posts test questions, observes results, identifies issues with students' learning, and adjusts the content of classroom teaching. This phase allows teachers to avoid a “one-size-fits-all” didactic teaching style in the process of knowledge imparting and to integrate the current stage of learning with the next stage of learning by understanding the current knowledge level of students so that the learning can be more targeted.

Phase 2: exploratory in-depth learning, focusing on students' acquisition of in-depth knowledge. Teacher and students enter the same video conference at the same time using the app “Tencent Meeting” and participate in at least one hour of intensive learning around the learning topic of the week. During such sessions, the teacher will make full use of conversation, scenarios, collaboration, and other elements to bring into play students' initiative and help them internalize their knowledge. The specific procedure is to analyze and review the common, complicated problems in the learning process based on the feedback from the prelesson test. The teacher then facilitates a heuristic dialogue session around the students' current problems and the week's learning topic. For example, based on the content of the learning topic, students are asked to think about why they want to learn about the topic and how the content is related to their work, and then, they are facilitated to discuss with other students the possible answers to questions raised based on their own work experience. In this phase of learning, students are familiar with the learning content and there are possibilities for them to explore, which can stimulate their initiative and motivation to participate in learning, as well as exercise their self-reflection and communication/expressing skills. It should be noted that the questions which the teacher gives to students at this stage are not related to the discussion in the forum at the next stage and students will not be encouraged to actively participate in the subsequent forum discussions.

Phase 3: reflective enhanced learning, focusing on students' acquisition of in-depth knowledge. In this phase, the teacher assigns forum discussion on the learning platform. The forum topics introduce case studies relevant to students' work through scenarios, and students are asked to leverage what they have learned in the week to analyze and solve the problems in the cases and propose recommendations for improvement. The forum activities require students to consolidate their knowledge through communication, collaboration, and sharing and learn to use their knowledge to solve actual problems. In this process, the teacher selects and edits the cases by referring to the knowledge covered in the previous phase. For example, if questions of “what? why?” are asked in the previous phase, the case should present the “what” and “why” in a story scenario so that students can apply their internalized knowledge. During the forum activities, teachers need to guide students to think and encourage their communication and collaboration.

#### 3.2.2. Design of Equal Group Experiment

Using the natural experiment method, the sample of 77 students was randomly divided into two groups, with 39 students in the experimental group implementing a flipped classroom-based approach and 38 students in the control group following the conventional approach. The difference between the two groups was primarily articulated in the sequence of teaching phases. Students in the experimental group were required to complete the learning tasks in the order of the three phases depicted in Figure 1, while students in the control group were free to choose the sequence of learning tasks according to their individual needs. Secondly, the teaching process in the second phase was different, as the experimental group was taught through exploratory and collaborative learning activities, while the control group was mainly taught by a teacher who directly imparted knowledge.

Both models were implemented online through the same app and course platform. The course resources, quiz questions, forum activities, and assessment criteria were the same for both groups, and they were taught by the same instructor. The instructor refers to the same teaching syllabus; that is, the teaching objectives and content of the two models are relatively consistent. The instructor used a “sandwich” approach to facilitate forum activities for both groups of students, first affirming students' responses, then proposing modifications, and finally encouraging students to browse other students' postings. The experimental environment was kept as consistent as possible. The experiment lasted for six weeks, i.e., from the beginning to the end of the course. The study was based on the following three hypotheses:

  H1: implementing an online flipped classroom helps improve students' capacity in cognitive processing
  H2: implementing an online flipped classroom helps to improve students' knowledge construction
  H3: implementing an online flipped classroom helps establish complicated knowledge network patterns

### 3.3. Data Analysis Tools and Methods

Since this study primarily examines social interactions in the context of asynchronous interactions, changes in students' cognition and construction levels are manifested in the quality of their cognitive processing process and the quality of meaning negotiation and knowledge coconstruction processes in the course forum, which can be measured by content analysis method. The student group knowledge network is manifested in the process of establishing close connections with peers, and the network density in the course forum can be measured by the social network analysis method. In the study, data analysis was performed to evaluate the quality of forum interactions by using SPSS 20.0 and NVivo 11.0, and UCINET 6.0 was used to measure the density of the forum network.

## 4. Experiment Results

### 4.1. Students' Cognitive Engagement Levels

The taxonomy of learning objectives proposed by American educator Benjamin Bloom defines the educational objectives in the cognitive domain as memorization, comprehension, application, analysis, evaluation, and creation. This represents the process of cognitive engagement from superficial to in-depth level and the process of cognition capacity development from low to high level. This study analyzes the content depth of student-student interaction texts to understand students' in-depth knowledge cognition capacity based on Bloom's taxonomy of learning objectives and drawing on the cognitive processing behavior coding framework of Chen Beilei et al. [26], as shown in Table 1. By uniformly coding the content text data posted by students in the case study activities in the course forum, the cognitive processing behavior score of individual student was obtained, which represented the highest cognition capacity reached by the content of his/her post, e.g., a student whose post reached the levels of memorization, comprehension, and analysis would have a score of 4. Each student's score on each cognitive processing behavior was calculated.

After independent sample *t*-tests were conducted on the cognitive processing behavior level scores of all students in the experimental and control groups, the results of the analysis are shown in Table 2.

As shown in Table 2, there was a statistically significant difference in cognition capacity between the experimental group and the control group (*p*=0.000 < 0.01). Specifically, under the influence of flipped classroom, the cognition capacity of the experimental group students was significantly higher than that of the control group students and H1 was verified.

**Figure 2:**
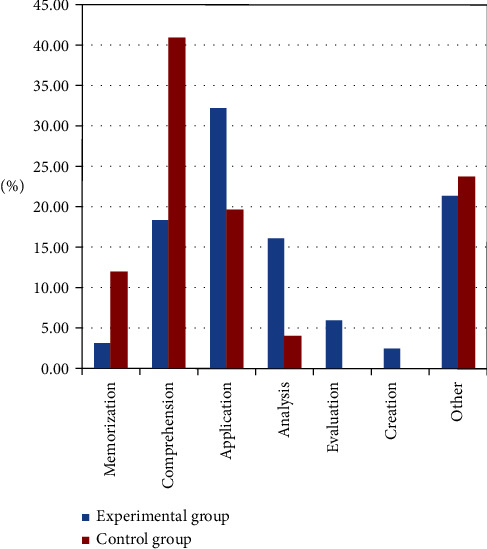
Characteristics of students' cognitive processing behaviors in the experimental and control groups.

Further coding of 501 post-based units at the cognitive structure level revealed that the proportion of the two superficial cognitive processing behaviors, i.e., memorization and comprehension, was 21.66%, and the proportion of the four in-depth cognitive processing behaviors, i.e., application, analysis, evaluation, and creation, was 56.95% among the students in the experimental group. In contrast, the proportions were 52.57% and 23.63%, respectively, among students in the control group. The proportion of interactive content not related to the topic in the experimental and control groups was 21.39% and 23.62%, respectively, as shown in Figure 2. This shows that the implementation of flipped classroom enables students not only to memorize and understand the factual attributes and conceptual information of knowledge but also to process it to a greater extent to solve real-world problems, develop a theoretical system by analyzing the problems and reflecting on the principles and values of knowledge, develop higher-order thinking skills such as critical thinking and creative thinking, and thus enhance the depth of cognition.

### 4.2. Students' Knowledge Construction Levels

The level of construction of student-student interaction is reflected by the phases of knowledge construction. In this study, the content text data of student postings in the case study activities in the course forum were coded uniformly based on Gunawardena's cue-based retrieval model [27], while considering the content meaning, i.e., there might be more than two codes for a posting. The codes were then matched with different interaction phases based on the content of the posting, the social construction level of each member was determined by the highest phase reached by the content of their interaction, and the construction levels were assigned the value of 1, 2, 3, 4, or 5 in a hierarchical order, from low to high, respectively [28]. An individual student's knowledge construction level score was the highest interaction phase he/she reached; e.g., a student whose posting content reached phase 1, phase 3, and phase 5 had a score of 5. The higher the student's score, the higher the quality of his/her meaning construction in that forum and hence the higher the level of construction. In this study, the content of interactions other than knowledge construction was uniformly coded as “other,” specifically, meaningless “like,” recognition, encouragement, and others towards others' posting, and was assigned a score of 0. See Table 3 for details.

Independent sample *t*-tests were conducted on all students' knowledge construction level scores in the experimental and control groups. The results of the analysis are shown in Table 4.

As shown in Table 5, there was a statistically significant difference in the level of construction between the experimental and the control group students (*p*=0.000 < 0.01). Specifically, under the influence of flipped classroom, the experimental group students had a significantly higher level of construction than the control group students and H2 was verified.

**Figure 3:**
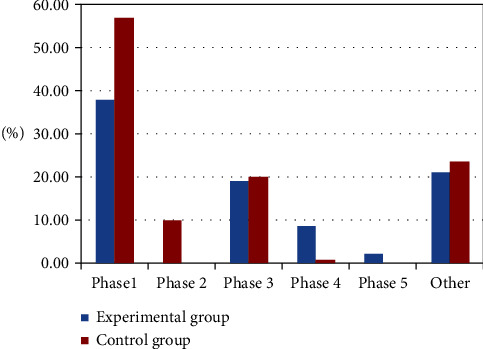
Characteristics of students' construction behaviors in the experimental and control groups.

Further coding of 501 post-based units at the construction phase level revealed that 48.13% of the students in the experimental group posted in the first and second phases of the low-level construction, with interactive information involving observations or opinion statements and questioning others' views. In contrast, the percentage of postings in the third, fourth, and fifth phases of high-level construction was 30.48%, and the interactive information involved expressing their own views or modifying views about others' opinions and updating their own views, and others. In the control group, the proportion of postings in the first and second phases was 56.70% and the proportion of postings in the third to fifth phases was 19.69%. The percentages of “other” postings in the two groups were 21.39% and 23.62%, respectively, as shown in Figure 3. These show that flipped classroom can help students learn the learning method of communication and collaboration with peers. In the process of knowledge construction, students can not only express their own views but also pay attention to others' views, make judgment, analysis, and reflection based on others' knowledge and experience, and then adjust their own conceptual system and experience system to achieve a meaningful construction process.

### 4.3. Students' Knowledge Network Patterns

Density is commonly measured to understand the density of social relationships and the evolution trend in online social networks, which is the ratio of the number of relationships actually present in the network to the maximum number of relationships that can be accommodated; the greater the overall network density, the stronger the connections between network members, i.e., the more complex the pattern of interaction behaviors [28]. In this study, data on the echo relations between students' postings in the course forum in the experimental and control groups were generated, and a relationship matrix was drawn. Based on the two interaction adjacency matrices obtained, a knowledge network diagram of student interactions in different teaching models was drawn using NetDraw, a visualization tool in UCINET. It should be noted that in this study, only postings with explicit echo targets were counted in the statistics, and postings without explicit echo targets or announcement-type postings targeting all forum members were not counted. The results of the analysis are shown in Table 5.

The results show that there were 39 students and 1 instructor in the course forum of the experimental group, and there were 428 pairs of relationships in the interaction process; the network witnessed 27.44% of network connections, and 21.3% of the connections were two-way interactions. In the control group, there were 38 students and 1 teacher in the course forum, and 158 pairs of relationships existed in the interaction process; the network witnessed 10.66% of network connections, and only 6.59% of the connections were two-way interactions. This shows that under the influence of flipped classroom, the knowledge network patterns formed among the members are more complicated, with stronger connectivity, suggesting that there are more knowledge innovation and better collective learning based on such network and H3 was verified.

**Figure 4:**
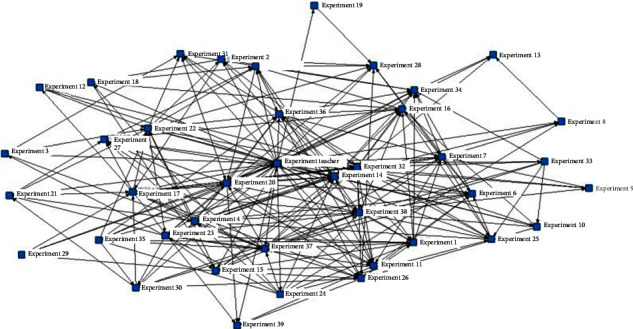
Social network diagram of the experimental group.

**Figure 5:**
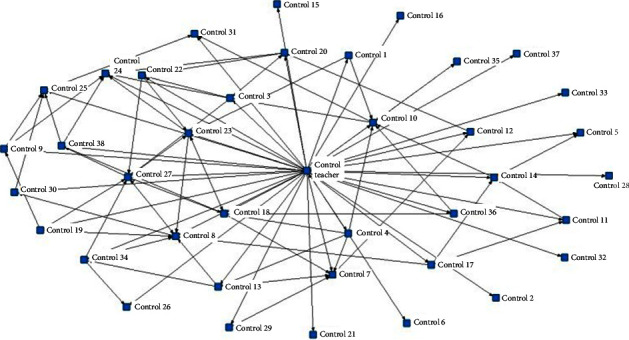
Social network diagram of the control group.

Further analysis of the community mapping and the individual degree centrality in the network revealed that the knowledge network pattern formed by the experimental group was more complicated, with stronger connectivity. The teacher and student nos. 32, 20, 7, 22, and 38 were at a more central position in the network and exhibited some initiative. The out-degree of the teacher was 140 and the in-degree was 16, indicating that the teacher gave feedback to almost all students' postings, and in turn, some students responded to the teacher's feedback. The means of individual out-degree and in-degree of other core members were 13 and 15, respectively, indicating that although the core members did not respond to each of their peers' postings as the teachers did, they were trying to follow and give feedback to peers and their behaviors increasingly resembled the “teacher role.” In the control group, only the teacher was in the central position and was the dominant player in the network. The teacher's out-degree was 94 and the in-degree was 2, suggesting that the teacher responded to students' postings, while the students seldom responded to the teacher. Other students had a mean in-degree of about 2 and a mean out-degree of 4, indicating that the students seldom interacted with each other and were simply used to answering questions around the teacher's topic postings. This shows that flipped classroom can draw students' attention to issues in their own learning and those of their peers so that they can go beyond the conventional “teacher-led” model and help each other to solve problems on their own. See Figure 4 and Figure 5 for details.

## 5. Analysis and Discussion

The experimental results show that online flipped classroom can effectively improve students' in-depth cognition capacity and lead to quality construction and more diversified knowledge network patterns, which can be attributed to the following.

### 5.1. Online Flipped Classroom Helps Develop Students' Higher-Order Thinking Capability and Mobilize In-Depth Cognitive Engagement

The experiment results showed that the cognition capacity of the students in the experimental group was significantly higher than that of the control group, and their cognitive behaviors were mostly at the “application and analysis” phases, while a few of them reached the “evaluation and creation” phases. In contrast, the cognitive behaviors of students in the control group were concentrated in the two superficial cognition phases of memorization and comprehension, and only a few of them reached the phases of “analysis and application”; rational analysis and multiperspective evaluation were generally absent in their application of knowledge. This suggests that conventional online teaching in open universities can hardly stimulate students' engagement with in-depth cognition. Conventional online teaching generally sets up free and flexible classroom sessions based on adult learning experiences, including viewing learning resources, participating in online live classes, completing discussion activities, and completing assignments. Teacher-student and student-student interactions usually occur in online live classes and discussion activities. However, the teaching during online live class is mostly composed of the teacher's lecture on knowledge. Moreover, in the process of such lecture, although the teacher will ask questions to mobilize students' thinking, the questions are mainly about factual knowledge and fail to mobilize students' higher-order capabilities such as critical thinking and creative thinking, so students' cognition remains at the phase of memorization and comprehension. This is the same as the cognitive processing the students implement in viewing learning resources, both of which simply involve the development of students' superficial cognition.

Online flipped classroom develops a three-phase process of “self-directed learning-exploratory learning-reflective learning” and designs classroom-based learning activities from superficial to in-depth levels at different phases. This helps students engage their thinking skills at each phase in a sequential manner. Firstly, the students view the learning resources to additionally remember and comprehend the knowledge and concepts they have already been exposed to, which stimulates the engagement of basic thinking. In an online live class, the instructor organizes exploratory thinking activities, facilitating students to retrieve the information they have viewed in the self-directed learning phase and to use higher-order thinking skills such as “application, analysis, and evaluation” to retrieve and summarize knowledge, thus establishing a unique knowledge system. Real-time interactive comments from peers and teachers also help students to constantly update their knowledge and experience and ensure cognitive validity. Such exploratory online live class provides a foundation for students to apply higher-order thinking skills in subsequent discussion forum activities. On the one hand, the students' cognition capacity has been raised to the “application, analysis, and even evaluation” phases in the online live class, so they can continue to think at the same level of cognitive engagement in the discussion forum. In addition, a few students are able to participate in the interactions with teachers and peers in the discussion forum to further improve and innovate their knowledge system and reach the highest cognition level. This step-by-step learning process helps adult students to logically sort out their fragmented knowledge and experiences through knowledge-imparting-style teaching, further enhance their construction in effective exploratory teaching, and eventually transfer and enhance them in interactive scenario-based teaching. This also confirms what has been mentioned in previous studies: students' higher-order thinking occurs after they exercise procedural knowledge and is enhanced through their self-directed exploration, thinking, and problem-solving under the guidance of the teacher [29]. The entire process of flipped classroom, from self-directed learning before class and exploratory learning based on learning activities during class to reflection and extension after class, can support students to achieve in-depth learning in a step-by-step manner, which is conducive to the development of students' higher-order thinking [29, 30].

### 5.2. Online Flipped Classroom Enhances Students' Sense and Behavior of Collaboration and Helps Achieve Collaborative Learning

According to the results of the experiment, after the implementation of flipped classroom, the level of social construction of the students in the experimental group was significantly higher than that of the students in the control group. In the low-level construction phase, 38% of the postings of the students in the experimental group were stating and sharing their own views and 10% were questioning others' views. In contrast, 57% of the control group's postings were stating and sharing their own views and no one challenged others' postings. In the high-level construction phase, 19% of the postings of the experimental group students were discussing with others to propose new views, 9% were revising their own views, and 2% applied the new views for construction. In contrast, 19% of the postings of control group students were proposing new views in response to others' postings, very few students revised their own views, and no students applied new views for construction. Respectively, 21.39% and 23.62% of the postings of the two groups were simply showing emotions. This shows that in conventional online teaching at open universities, students believe that the main tasks in discussion activities are to post and share their views and that collaboration simply involves telling others about their postings in the form of replies or replying to postings that show emotions. These further show that the students are not aware of what is a meaningful act of constructing or the method of constructing.

In online flipped classroom, students' collaborative learning is reflected in two teaching sessions: online live class and discussion forum. The online live class is a type of synchronous, interactive, exploratory learning process, in which the teacher first summarizes the issues with students' tests and discusses with the class the questions and challenges in learning, facilitating students to ask questions, analyze, integrate, reflect, and communicate by referring to their existing knowledge and experience. The exploratory process not only deepens students' understanding and views of the issues but also develops students' ability to express, communicate, and exchange and focuses on the methods they learn in the process of constructing knowledge. As a result, students learn to apply the relevant collaboration methods in subsequent forum activities and are able to revise and even reconstruct the questions through communication and reflection, thus improving their level of construction. This further confirms what has been mentioned in previous studies: flipped classroom can significantly improve the problem that students' cooperative learning is a mere formality, and the interactive, cooperative, and participatory nature of flipped classroom makes it necessary for students to collaborate with each other to complete the learning, which promotes the development of students' collaboration skills and thus ensures the quality of learning [31, 32].

### 5.3. Online Flipped Classroom Stimulates Students' Initiative and Contributes to the Generation of Knowledge Innovation

According to the experimental results, in flipped classroom model, members have closer relationships with each other, participate more interactively in a forum, and exhibit more personal contributions. In comparison, although conventional online teaching provides learners with open online learning resources and advocates that adult learners are “teachers” with experience, the process of teaching implementation remains at the level of a “teacher-centered” approach; i.e., the teacher leads the entire teaching process and students follow the “command” to complete the learning. Although such open forum activities give students space for independent exploration, the students simply follow the teacher's instructions to answer the test questions and attend online live classes, with limited self-efficacy and hence no desire to show themselves in the forum activities. These result in limited interactive participation of students, which is not conducive to the “collision” of ideas and generation of new knowledge. This is consistent with what has been mentioned in other studies: adult learners are influenced by conventional education and are accustomed to simply listening and receiving information in online learning, which leads to their low motivation for raising questions and sharing answers in the learning process and limited ability to explore knowledge in depth [33, 34]. In addition, because there is basically no communication among students prior to forum activities, the emotional linkages are weak, and therefore, they are reluctant to point out the shortfalls of others in the forum activities and lack two-way communication. This confirms what has been mentioned in other studies: students are reluctant to communicate with people they are not familiar with in online learning and therefore two-way interaction is limited [10].

Flipped classroom is consistently based on the “student-centered” approach, in which the teacher is merely the organizer and facilitator of teaching activities. Therefore, online flipped classroom organizes online live class activities targeting the issues exhibited by students in their self-directed learning and designs exploratory activities focusing on learners' initiative actions, which is conducive to stimulating students' interests and experiences to participate in the construction of knowledge. Firstly, online live class is centered on the problems that students face, so that students gain stronger ownership, showcase themselves through dialogue, reflection, and communication to solve problems in group activities, enhance their self-confidence, and establish emotional connections. As a result, in subsequent forum activities, students' learning potential and motivation will be stimulated, and they will be able to express their views courageously, identify issues proactively, and contribute their knowledge and power in order to better present themselves in an interpersonal environment more familiar to them. In this process, teaching and learning are no longer one-to-one but many-to-many; i.e., each student has the confidence, ability, and emotional basis to disagree with and revise others' postings as a “teacher,” while accepting others' suggestions as a student and working together to improve and revise answers, thus realizing knowledge innovation. This also confirms what has been mentioned in other studies: the speech behavior of students in the traditional mode is mainly reflected in the passive response of students to teachers. In the flipped classroom mode, students use more time to share their knowledge achievements and play the role of teachers. It can stimulate students' learning motivation and potential, make them actively participate in learning, gain a better sense of self-efficacy through self-exploration, and finally enhance learning effectiveness [35, 36].

## 6. Study Limitations and Outlook

This study developed an online teaching model based on flipped classroom, which is designed to promote students' interactive participation in online learning and enhance the quality of interactions by leveraging the advantages of flipped classroom. In order to evaluate the actual effect of the teaching model, an empirical analysis was conducted from three perspectives: students' cognitive participation level, knowledge construction level, and knowledge network patterns. Although the effectiveness of online flipped classroom was confirmed, the study came with several limitations. First, the design of the online teaching model based on flipped classroom is still immature. Therefore, the researchers were prudent in defining the scope of the experiment and designed and studied only two weeks of course learning which covered a short period of time and a limited number of forum activities. Hence, the validity of the experimental results will still need to be established in the future through large-scale and long-term validation. Second, this study selected adult students majoring in preschool education as the data source, and the sample was not adequately representative; whether there are differences among students of different majors remains to be examined. Third, constrained by the length of the paper, although this study explored the changes in student interaction behaviors and interaction quality, it fails to address the influence of teachers in this process [37].

Therefore, the following recommendations are proposed for subsequent future studies: (1) continue to explore the cognitive characteristics of students in flipped classroom-based online teaching, and examine the impact of online flipped classroom on students' in-depth cognition from two dimensions: objectives and outcomes, (2) focus on the influence and role of teachers in flipped classroom, and analyze how interactive teaching activities are carried out between teachers and students, and (3) examine the interaction behaviors and quality of interactions among different types of learners in flipped classroom, and conduct an empirical study on improvement to the teaching effectiveness from the perspective of social division of labor.

## Figures and Tables

**Table 1:** Cognitive processing behavior coding framework.

Indicator for coding	Description	Score
Memorization	Retrieval of relevant knowledge or facts from long-term memory	1
Comprehension	Apprehension of knowledge, relating new knowledge to existing knowledge or previous experiences, and constructing the meaning	2
Application	Application in order to complete a task or solve a problem in a scenario	3
Analysis	Break down knowledge into its parts, analyze each part, and reveal the relationship between each part and the whole	4
Evaluation	Make judgments or comments based on certain principles and criteria	5
Creation	Combine different elements and create a complete, functional whole, and reorganize the elements into a new pattern or structure through the thinking process	6
Other	Interactive content not related to the topic	0

**Table 2:** Comparison of cognition capacity of the experimental group and the control group.

Dimension	Variable	Group	Sample size	Mean	Standard deviation	*t*
Cognition capacity	Flipped classroom	Experimental group	39	3.5385	1.3148	5.065^*∗∗*^
		Control group	38	2.2105	0.9630	

Note: ^*∗*^*p* < 0.05; ^*∗∗*^*p* < 0.001.

**Table 3:** Coding based on Gunawardena's cue-based retrieval model.

Interaction phase	Indicator for interaction content coding	Score
Phase 1	Present and share views	1
Phase 2	Express disagreement	2
Phase 3	Discuss and present new views	3
Phase 4	Test and revise views	4
Phase 5	Members reach a consensus and apply new constructs	5
Other	Meaningless “like” or recognition	0

**Table 4:** Comparison of the level of knowledge construction between the experimental group and the control group.

Dimension	Variable	Group	Sample size	Mean	Standard deviation	*t*
Construction level	Flipped classroom	Experimental group	39	3.0256	1.49538	4.526^*∗∗*^
		Control group	38	1.7059	0.97014	

Note: ^*∗*^*p* < 0.05; ^*∗∗*^*p* < 0.01.

**Table 5:** Social network attributes of students in the experimental and control groups.

Teaching model	Network density		Number of nodes	Number of connections	Reciprocity (%)
	Mean	Standard deviation			
Experimental group	0.2744	0.7674	40	428	21.3
Control group	0.1066	0.4977	39	158	6.59

## Data Availability

The datasets used during the current study are available from the corresponding author upon reasonable request.
